# Comparison of LDH activity and LDH-M to predict death in patients with acetaminophen-induced acute liver failure

**DOI:** 10.17179/excli2025-9174

**Published:** 2026-02-04

**Authors:** Debasree Bhadra, Jody A. Rule, William M. Lee, Mitchell R. McGill

**Affiliations:** 1 Dept. of Pharmacology and Toxicology, College of Medicine, University of Arkansas for Medical Sciences, Little Rock, AR USA; 2 Div. of Digestive and Liver Diseases, Dept. of Internal Medicine, UT Southwestern Medical Center, Dallas, TX USA; 3 Dept. of Environmental Health Sciences, Fay W. Boozman College of Public Health, University of Arkansas for Medical Sciences, Little Rock AR USA; 4 Dept. of Pathology, College of Medicine, University of Arkansas for Medical Sciences, Little Rock, AR USA

**Keywords:** biomarker, drug-induced liver injury, hepatotoxicity, prognosis

## Abstract

Liver transplantation is often the only life-saving intervention in acute liver failure (ALF), so it is critical to identify all ALF patients who need a transplant to survive. However, transplantable organs are scarce and recipients face significant life-threatening risks, so it is also important to avoid transplantation when possible. Thus, prognostics that can predict death in ALF with both high sensitivity and specificity are needed. We previously demonstrated total lactate dehydrogenase (LDH) activity may be useful for this purpose. However, LDH is a tetramer of LDH-M and LDH-H, and proteomics indicated only the liver enzyme - LDH-M - increases in non-survivors. LDH-H decreased. Because total LDH comprises both, it is possible that LDH-M could have even better prognostic performance. To test that, we compared total LDH and LDH-M in a subset of samples from our prior study of survivors and non-survivors of acetaminophen (APAP)-induced ALF. The model for end-stage liver disease (MELD) score and the MELD-LDH score were also calculated. Both total LDH and LDH-M values were greater in non-survivors than survivors, but there was no significant difference in prognostic metrics between them. Overall, total LDH performed similarly to or modestly better than both LDH-M and the MELD. The MELD-LDH score also performed somewhat better than the MELD.

See also the graphical abstract(Fig. 1).

## Introduction

Acute liver failure (ALF) is a rare but dire condition characterized by rapid deterioration of hepatic function secondary to acute liver injury. Incidence varies between countries but is typically in the range of 1-15 cases per million persons per year (McGill, 2025[7]). Despite its relatively rare occurrence, however, it can be catastrophic - mortality is in the range of 20-40 % in the US and similar countries and even higher elsewhere (Reuben et al., 2016[13]; Thanapirom et al., 2019[14]; McGill, 2025[7]).

New prognostics could improve management of ALF patients. An ideal prognostic would have high sensitivity for death to identify all patients in need of a liver transplant for survival. On the other hand, there is often a scarcity of transplantable livers and recipients face significant risks that can also lead to death (Putignano et al., 2018[12]; Karvellas et al., 2023[4]; Moughames et al., 2024[10]). In addition, liver transplantation is expensive (Ufere et al., 2024[15]). For these reasons, an ideal prognostic would also have high specificity to avoid transplantation in patients who can recover without it. Current prognostic indicators fall short. Some have high specificity but low sensitivity (e.g. the King's College Criteria [KCC]) while others usually have moderate values for both (e.g. the Model for End-stage Liver Disease [MELD] score) (McGill, 2025[7]). In addition, these prognostics consist of multiple laboratory values and/or clinical observations, which increases complexity and opportunities for error (McGill, 2025[7]). A single biomarker is likely preferable.

In a previous study, our analysis of serum from survivors and non-survivors of acetaminophen (APAP)-induced ALF revealed that total lactate dehydrogenase (LDH) activity can predict death and therefore the need for a transplant (Vazquez et al., 2022[18]). We then verified those results in a larger population with ALF that included other etiologies (Price et al., 2023[11]). Interestingly, however, LDH comprises five tetramers (LDH1-5) consisting of two repeating subunits, LDH-M and LDH-H, that are encoded by separate genes, *LDHA* and *LDHB*, and our proteomics data indicated that only LDH-M increased in non-survivors (Vazquez et al., 2022[18]; Price et al., 2023[11]). Based on that observation, we hypothesized that LDH-M is a better prognostic biomarker in ALF than total LDH, which is likely a weighted average of the five tetramers containing both enzymes. In the present study, we tested that hypothesis by comparing serum LDH-M concentration and total LDH activity in a subset of samples from our earlier study of APAP-induced ALF patients (Vazquez et al., 2022[18]). Both metrics were also compared to the MELD score as a common benchmark. Finally, the MELD-LDH score was also calculated and compared with LDH and the standard MELD score.

## Materials and Methods

### Human subjects approval

Samples, patient demographics, and clinical laboratory data were obtained from the Acute Liver Failure Study Group (ALFSG) biorepository. Internal review board (IRB) approval was obtained at each ALFSG study site and the study was conducted in accordance with the 1975 Declaration of Helsinki. Written informed consent was obtained from next of kin.

### Patient samples

This study analyzed residual human serum samples from a larger set described in our prior work (Vazquez et al., 2022[18]). The subset consisted of samples from 21 transplant-free survivors and 18 non-survivors of APAP-induced ALF that were collected on day 3 of admission to the ALFSG. Day 3 was chosen because it is near the time of peak injury and peak LDH (Price et al., 2023[11]). The diagnosis of APAP-induced ALF was made by ALFSG site investigators. Diagnostic criteria included alanine aminotransferase (ALT) ≥ 1,000 U/L, illness duration less than 26 weeks, hepatic encephalopathy, coagulopathy (INR ≥ 1.5), and absence of chronic liver disease. Serum was separated by centrifugation at each ALFSG site and stored at −80 °C for later distribution and use.

### Clinical biochemistry

ALT, bilirubin (Bili), prothrombin time (PT), and international normalized ratio (INR) were measured at ALFSG study sites using standard clinical laboratory methods. To determine LDH activity in our laboratory, a kinetic assay that measures the loss of reduced nicotinamide adenine dinucleotide (NADH) absorbance at 340 nm in the reaction mixture was used as described previously (Vazquez et al., 2022[18]). LDH-M concentration was measured using a commercially available human LDHA ELISA kit from LSBio (Cat. No. LS-F4579, Newark, CA, USA), according to the manufacturer's instructions. Lack of cross-reactivity with LDH-H/LDHB and with other lactate dehydrogenases was confirmed by the manufacturer (personal communication). MELD score was calculated as MELD = 9.6×ln(Creatinine) + 3.8×ln(bilirubin) + 11.2×ln(INR) + 6.4. The MELD-LDH score was calculated as MELD-LDH = -5.844 + 0.682×log(LDH) + 2.702×log(MELD).

### Statistics

Mean results from survivors and non-survivors were compared using the Mann-Whitney U-test in Prism v10.0 (GraphPad Software, Boston, MA). Receiver operating characteristic (ROC) curve analyses were performed in R (R Foundation for Statistical Computing, Vienna, AUT) using the fbroc package with 1,000 bootstrap resamples. The 95 % confidence intervals (CIs) for sensitivity and specificity were calculated by bootstrapping with 1,000 resamples using custom scripts in R. Either McNemar's test or a two-sample z-test was performed to determine if the differences in sensitivity or specificity between LDH, LDH-M, or the MELD-LDH and the MELD score were significant (p < 0.05).

## Results

**Figure 2:**
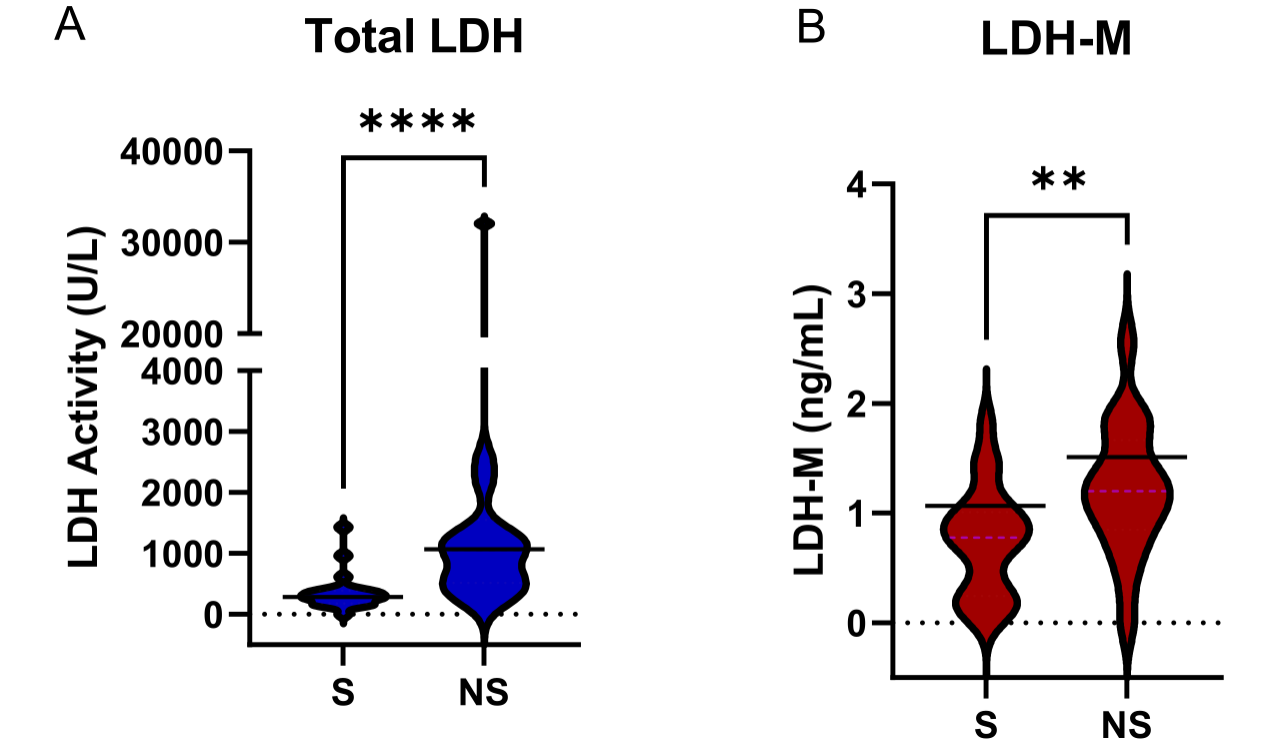
Total LDH activity and LDH-M concentration in serum samples from APAP-induced ALF patients. Serum samples from survivors (S) and non-survivors (NS) of APAP-induced ALF collected on day 3 of study admission were acquired from the Acute Liver Failure Study Group biorepository. (A) Total lactate dehydrogenase (LDH) activities. (B) LDH-M concentrations. Data are expressed as violin plots. Solid lines represent mean values. **p < 0.01. ****p < 0.0001.

Patient demographics and laboratory values are provided in Table 1(Tab. 1) and Supplementary Table 1. Consistent with our earlier work, total LDH activity was significantly elevated in serum from non-survivors compared to survivors (Figure 2A(Fig. 2)). Importantly, LDH-M was similarly elevated in non-survivors (Figure 2B(Fig. 2)).

**Figure 3:**
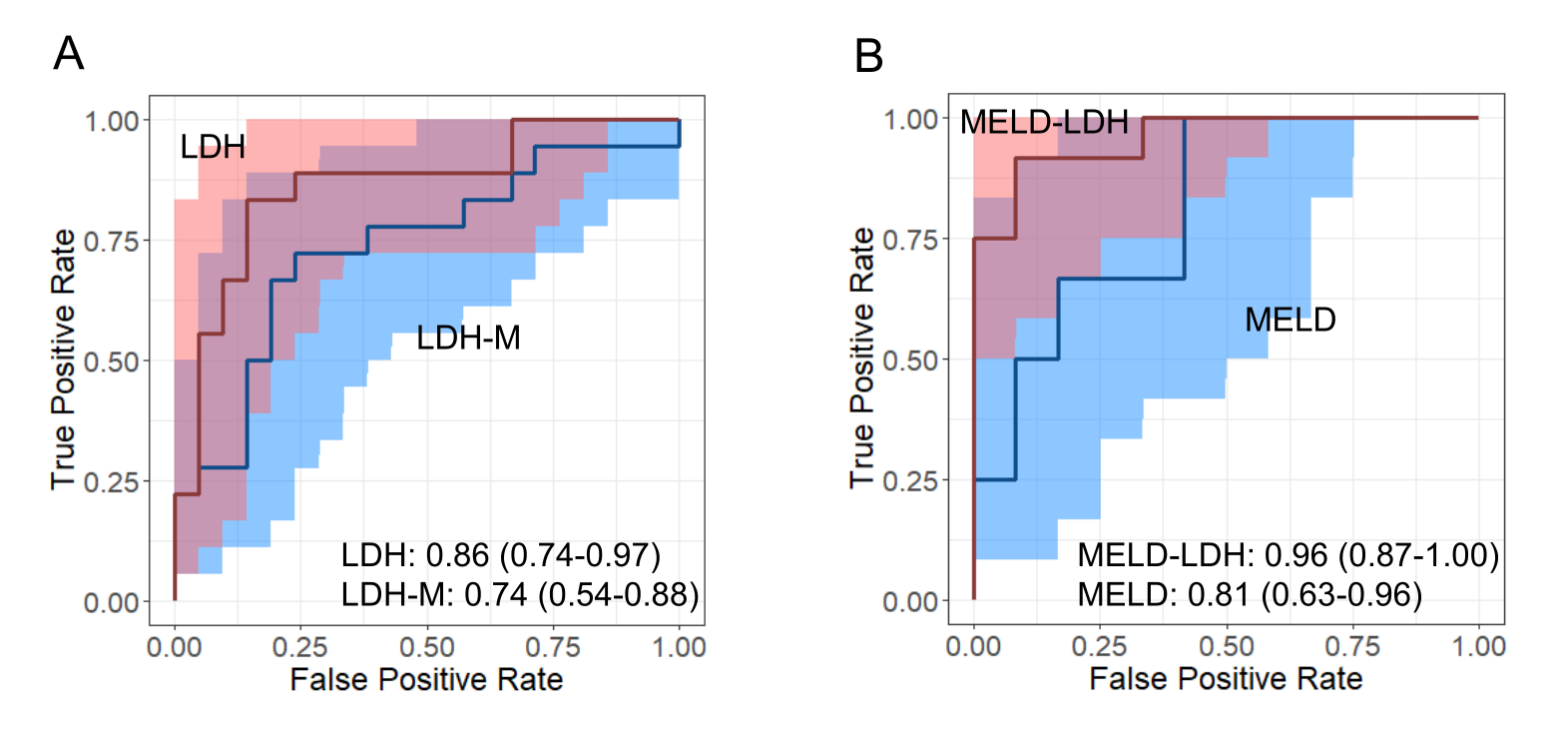
Ability of total LDH, LDH-M, and the MELD score to predict death. Receiver operating characteristic (ROC) curves for death for (A) total lactate dehydrogenase (LDH) (red) and LDH-M (blue) and (B) the Model for End-stage Liver Disease (MELD) score. Shaded areas show the 95 % confidence intervals (95 % CIs) around the lines. Area under the curve values with 95 % CIs are at the bottom of each panel. Note that the MELD and MELD-LDH scores could only be calculated for 24 of the 39 patients.

To compare the prognostic value of total LDH and LDH-M, we performed ROC curve analyses. Both biomarkers were significantly associated with death (Figure 3A(Fig. 3)), but there was no difference between them. Their areas under the curve (AUCs) for both markers were also similar to the MELD score (cutoff: 25) (Figure 3B(Fig. 3)). Interestingly, the AUC for the MELD-LDH score outperformed all three (p-values in the range of 0.010 to 0.023 for the comparisons). We then determined the sensitivities and specificities of these biomarkers for death (Table 2(Tab. 2)) at values greater than Youden's Index J (LDH-M: 0.978 ng/mL; LDH: 456 U/L; MELD-LDH: 0.1). There were no major differences between LDH, LDH-M, and the MELD-LDH score. However, the specificities of total LDH and the MELD-LDH were greater than the specificity of the MELD score (p < 0.05). While the MELD tended to have greater sensitivity than any other marker in this cohort, the differences in sensitivity between total LDH and the MELD score and the MELD-LDH and the MELD score were not significant. Overall, the MELD-LDH had the greatest accuracy, followed by total LDH activity (Table 2(Tab. 2)).

**Figure 4:**
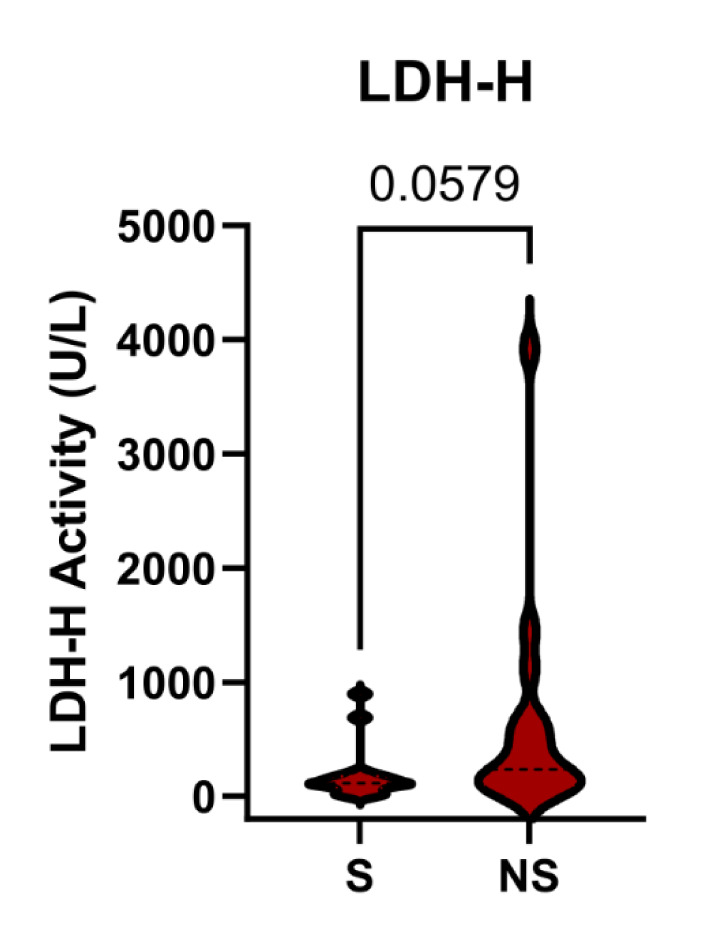
LDH-H activity in serum from APAP-induced ALF patients. Serum samples from survivors (S) and non-survivors (NS) of APAP-induced acute liver failure (ALF) collected on day 3 of study admission were acquired from the Acute Liver Failure Study Group biorepository. Lactate dehydrogenase H (LDH-H) activities were measured. Data are expressed as violin plots. The p-value is shown.

The finding that LDH-M performed like total LDH was consistent with our null hypothesis. One possible explanation for this result is that LDH-H is also elevated in non-survivors, contrary to our earlier observation (Vazquez et al., 2022[18]; Price et al., 2023[11]). To test that possibility, we measured LDH activity using a method known to favor LDH-1 (Bais and Philcox, 1994[2]), the tetramer composed primarily of LDH-H. Indeed, the results indicated that LDH-H is also elevated in non-survivors (Figure 4(Fig. 4)). The discrepancy between these data and our earlier proteomics data may be due to either the smaller sample size that we used for proteomics or a difference in timing, as the samples in the prior work were from day 1 of ALFSG study admission instead of day 3. In either case, both LDH-M and LDH-H are elevated in non-survivors compared to survivors overall.

## Discussion

The results from this study once again support the prognostic utility of circulating LDH activity and the MELD-LDH score in ALF. We initially identified LDH as a prognostic biomarker in 58 patients with APAP-induced ALF (Vazquez et al., 2022[18]). We then confirmed that observation through review of 170 patients with confirmed ALF of multiple etiologies and an additional 68 with biochemical evidence of ALF (238 patients total) (Price et al., 2023[11]). Altogether, the data demonstrate that total LDH activity is a single biomarker that works about as well as, or somewhat better than, the MELD to predict death in ALF patients. It also has the advantage of being widely available in clinical laboratories. There is also some indication that the MELD-LDH score may be even more useful than either LDH or the MELD score alone for prognosis based on the ROC AUC.

Our prior proteomics data indicated that LDH-M may work better than total LDH for ALF prognosis. In that study, we observed that LDH-M increased in serum from non-survivors while LDH-H decreased (Vazquez et al., 2022[18]; Price et al., 2023[10]). Because total LDH is essentially a weighted average of the two enzymes, we reasoned that the decrease in LDH-H could have suppressed the prognostic utility of total LDH such that LDH-M alone would work even better. The data from the present work indicate otherwise. LDH-M measured by ELISA performed no better than total LDH activity. In fact, we saw an increase in LDH-1 activity (which consists primarily of LDH-H) in non-survivors, like total LDH.

Importantly, the association between LDH activity and death appears robust to methodology. In our initial study of APAP-induced ALF patients (Vazquez et al., 2022[18]) and in the present study, we used a method that monitors NADH disappearance due to the LDH-catalyzed reduction of pyruvate to lactate at neutral pH. On the other hand, the method used in our hospital in our prior follow-up study (Price et al., 2023[11]) is based on the more popular International Federation of Clinical Chemistry (IFCC) method that measures NADH formation due to the LDH-catalyzed oxidation of lactate to pyruvate at pH 9.4. In all studies, LDH activity was predictive of death about as well as the MELD score. The use of different methods as well as different ALF etiologies between the two studies may also account for the difference in cutoff (Youden's Index J) between them. In this study and in our prior work with the larger group of APAP patients (Vazquez et al., 2022[18]; unpublished data), the optimal cutoff for total LDH was around 450-500 U/L. However, in our study with more etiologies and using the IFCC method, the optimal cutoff was around 2,000 U/L (Price et al., 2023[11]). Thus, the methodology and patient population should be considered when deciding on a prognostic threshold in future work.

It is not clear why LDH is associated with death in ALF while some enzymes like ALT are not. We previously ruled out that LDH is a marker of multi-organ damage despite its ubiquitous expression because our proteomics data showed a decrease in LDH-H - the non-liver form of LDH - in non-survivors (Price et al., 2023[11]). However, with the results from the present study, we can no longer rule out that hypothesis. Total LDH may indeed be a marker of multi-organ injury secondary to ALF. Importantly, multi-organ failure is a major cause of death in ALF patients (Reuben et al., 2016[13]) so that could explain why LDH is prognostic. In the context of APAP-induced ALF, specifically, renal damage is a predictor of poor outcomes (Antoine et al., 2015[1]), and serum LDH is elevated by kidney injury (Green et al., 2017[3]), so nephrotoxicity could be part of the reason for the association between LDH and death. Consistent with that, serum creatinine was higher in non-survivors here (Table 1(Tab. 1)). Another possible explanation is increased hepatocellular expression of LDH during acute liver injury. Consistent with that hypothesis, increased LDH staining has been reported in liver sections from ALF patients (Kotoh et al., 2011[5]). It is also possible that there is simply more LDH within hepatocytes than there is ALT such that ALT release is exhausted in non-survivors while LDH release continues, leading to greater LDH levels in blood when injury is worse. In any case, it is apparent that LDH and the MELD-LDH score are useful prognostics in ALF.

Importantly, this study has notable limitations. The study population was relatively small. A larger study could yield different results for LDH-H. In addition, the study was limited to APAP-induced ALF. The results for LDH-H and the comparison of LDH-H with LDH-M and the MELD score may differ for other etiologies. Additional research would be helpful to address these issues.

Overall, serum LDH and the MELD-LDH score are promising prognostic biomarkers in ALF, regardless of the method of LDH measurement. Future studies should further verify the utility of LDH and determine consistently effective cutoff values across ALF etiologies. It could also be interesting to test the combination of LDH with other prognostic markers, like alpha-fetoprotein (McGill and Jaeschke, 2018[8]), glutamate dehydrogenase (McGill et al., 2014[9]), certain bile acids (Woolbright et al., 2014[19]), CXCL14 (Umbaugh et al., 2024[16]), angiopoietin-2 (Umbaugh et al., 2025[17]), carbamoyl phosphate synthetase 1 (Kwan et al., 2023[6]), and several others (McGill and Jaeschke, 2018[8]; McGill, 2025[7]).

## Declaration

### Conflict of interest

In the last five years, MRM has consulted for Alkermes, GSK, Haleon, and Acetaminophen Toxicity Diagnostics (ATD), LLC, and has been an expert in litigation involving Kenvue. He has also received research funding from GSK and Haleon. None of these organizations had any role in the conception, design, or execution of these studies, nor in the preparation of the manuscript or decision to publish.

### Funding

The authors were supported in part by US National Institutes of Health grants 1R01DK135752 (to MRM) and U01DK058369 (to WML).

### Acknowledgments

We thank all ALFSG site investigators, staff, and participants.

### Artificial Intelligence (AI) - assisted technology

Artificial intelligence was not used in the preparation of this manuscript.

## Supplementary Material

Supplementary information

## Figures and Tables

**Table 1:**
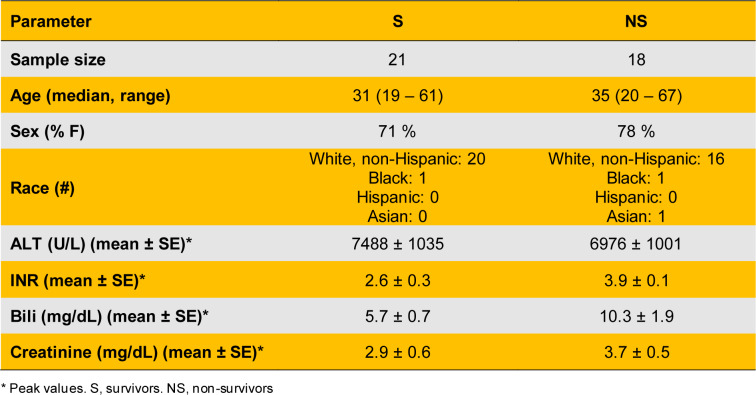
Patient demographics and clinical laboratory values

**Table 2:**
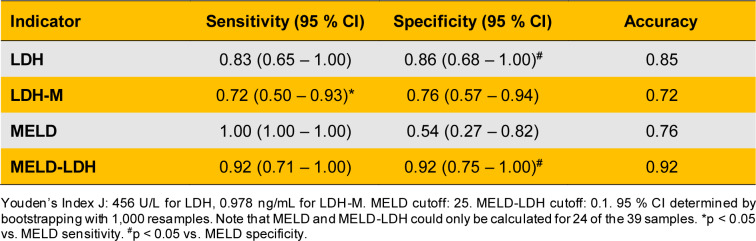
Clinical performance metrics

**Figure 1:**
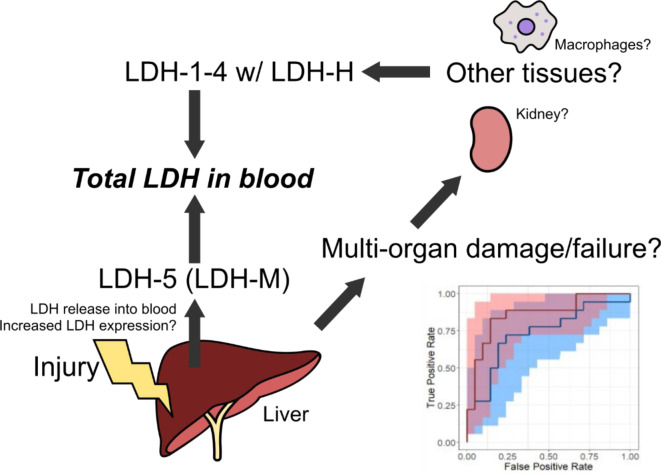
Graphical abstract

## References

[1] Antoine DJ, Sabbisetti VS, Francis B, Jorgensen AL, Craig DG, Simpson KJ (2015). Circulating kidney injury molecule 1 predicts prognosis and poor outcomes in patients with acetaminophen-induced liver injury. Hepatology.

[2] Bais R, Philcox M (1994). IFCC methods for the measurement of catalytic concentration of enzymes. Part 8. IFCC method for lactate dehydrogenase (L-lactate: NAD oxidoreductase, EC 1.1.1.27). J Automat Chem.

[3] Green H, Tobar A, Gafter-Gvili A, Leibovici L, Klein T, Rahamimov R (2017). Serum lactate dehydrogenase is elevated in ischemic acute tubular necrosis but not in acute rejection in kidney transplant patients. Prog Transplant.

[4] Karvellas CJ, Leventhal TM, Rakela JL, Zhang J, Durkalski V, Reddy KR (2023). Outcomes of patients with acute liver failure listed for liver transplantation: A multicenter prospective cohort analysis. Liver Transplantation.

[5] Kotoh K, Kato M, Kohjima M, Tanaka M, Miyazaki M, Nakamura K (2011). Lactate dehydrogenase production in hepatocytes is increased at an early stage of acute liver failure. Exp Ther Med.

[6] Kwan R, Chen L, Park MJ, Su Z, Weerasinghe SVW, Lee WM (2023). The Role of Carbamoyl Phosphate Synthetase 1 as a Prognostic Biomarker in Patients With Acetaminophen-induced Acute Liver Failure. Clin Gastroenterol Hepatol.

[7] McGill MR (2025). From fructose to the future: liver disease biomarkers and their prognostic value in acute liver failure. Crit Rev Clin Lab Sci.

[8] McGill MR, Jaeschke H (2018). Biomarkers of drug-induced liver injury: progress and utility in research, medicine, and regulation. Expert Rev Mol Diagn.

[9] McGill MR, Staggs VS, Sharpe MR, Lee WM, Jaeschke H (2014). Serum mitochondrial biomarkers and damage-associated molecular patterns are higher in acetaminophen overdose patients with poor outcome. Hepatology.

[10] Moughames E, Gurakar M, Khan A, Alsaqa M, Ozturk NB, Bonder A (2024). Recipient Survival among Living Donor vs. Deceased Donor Liver Transplants for Acute Liver Failure in the United States. J Clin Med.

[11] Price JR, Hagrass H, Filip AB, McGill MR (2023). LDH and the MELD-LDH in Severe Acute Liver Injury and Acute Liver Failure: Preliminary Confirmation of a Novel Prognostic Score for Risk Stratification. J Appl Lab Med.

[12] Putignano A, Figorilli F, Alabsawy E, Agarwal B, Jalan R (2018). Long‐term outcome in patients with acute liver failure. Liver International.

[13] Reuben A, Tillman H, Fontana RJ, Davern T, McGuire B, Stravitz RT (2016). Outcomes in Adults With Acute Liver Failure Between 1998 and 2013: An Observational Cohort Study. Ann Intern Med.

[14] Thanapirom K, Treeprasertsuk S, Soonthornworasiri N, Poovorawan K, Chaiteerakij R, Komolmit P (2019). The incidence, etiologies, outcomes, and predictors of mortality of acute liver failure in Thailand: a population-base study. BMC Gastroenterol.

[15] Ufere NN, Serper M, Kaplan A, Horick N, Indriolo T, Li L (2024). Financial burden following adult liver transplantation is common and associated with adverse recipient outcomes. Liver Transpl.

[16] Umbaugh DS, Nguyen NT, Curry SC, Rule JA, Lee WM, Ramachandran A (2024). The chemokine CXCL14 is a novel early prognostic biomarker for poor outcome in acetaminophen-induced acute liver failure. Hepatology.

[17] Umbaugh DS, Nguyen NT, Curry SC, Rule JA, Lee WM, Ramachandran A (2025). The endothelial growth factor angiopoietin-2 is an accurate prognostic biomarker in patients with acetaminophen-induced acute liver failure. Toxicol Sci.

[18] Vazquez JH, Kennon-McGill S, Byrum SD, Mackintosh SG, Jaeschke H, Williams DK (2022). Proteomics Indicates Lactate Dehydrogenase Is Prognostic in Acetaminophen-Induced Acute Liver Failure Patients and Reveals Altered Signaling Pathways. Toxicol Sci.

[19] Woolbright BL, McGill MR, Staggs VS, Winefield RD, Gholami P, Olyaee M (2014). Glycodeoxycholic acid levels as prognostic biomarker in acetaminophen-induced acute liver failure patients. Toxicol Sci.

