# Supplementary material for: Comparison of LDH activity and LDH-M to predict death in patients with acetaminophen-induced acute liver failure
Source: EXCLI J. 2026 Feb 4;25:330–8. doi: 10.17179/excli2025-9174 (PMC12982867; doi:10.17179/excli2025-9174)
Supplement: Supplementary information [file EXCLI-25-330-s-001.pdf]

EXCLI Journal 2026;25:330-338 – ISSN 1611-2156
Received: December 03, 2025, accepted: January 11, 2026, published: February 04, 2026

Supplementary information to:

Original article:

COMPARISON OF LDH ACTIVITY AND LDH-M TO PREDICT DEATH IN PATIENTS WITH ACETAMINOPHEN-INDUCED ACUTE LIVER FAILURE

Debasree Bhadra[1], Jody A. Rule[2], William M. Lee[2], Mitchell R. McGill[1,3,4*]; Acute Liver Failure Study Group

[1] Dept. of Pharmacology and Toxicology, College of Medicine, University of Arkansas for Medical Sciences, Little Rock, AR USA
[2] Div. of Digestive and Liver Diseases, Dept. of Internal Medicine, UT Southwestern Medical Center, Dallas, TX USA
[3] Dept. of Environmental Health Sciences, Fay W. Boozman College of Public Health, University of Arkansas for Medical Sciences, Little Rock AR USA
[4] Dept. of Pathology, College of Medicine, University of Arkansas for Medical Sciences, Little Rock, AR USA

* **Corresponding author:** Mitchell R. McGill, PhD, NRCC, FADLM, Associate Professor, University of Arkansas for Medical Sciences, 4301 W. Markham St., Slot 820, Little Rock, AR 72205 USA; E-mail: mmcgill@uams.edu

https://dx.doi.org/10.17179/excli2025-9174

This is an Open Access article distributed under the terms of the Creative Commons Attribution License (https://creativecommons.org/licenses/by/4.0/).

EXCLI Journal 2026;25:330-338 – ISSN 1611-2156
Received: December 03, 2025, accepted: January 11, 2026, published: February 04, 2026

Supplementary Table 1: Detailed patient information

Code	ALT (U/L)	Bilirubin (mg/dL)	Creatinine (mg/dL)	INR	Sex (1= male, 2 = female)	Ethnicity (2 = Non-Hispanic)	Race (1 = White, 2 = Black, 4 = Asian)	Age	Died? (1 = No, 2 = Yes)
1	7120	7.8	4.69	2	2	2	1	46	1
2	14725	13.7	7.3	4	1	2	2	29	1
3	5637	6.8	1.4	1	2	2	1	31	1
4	3194	11.1	2.7	2	2	2	1	31	1
5	6642	1.4	0.7	4	1	2	1		1
6	3388	5.5	0.8	4	2	2	1	37	1
7	7260	3.1	1.8	.	2	2	1	47	1
8	23700	2.6	0.6	3	2	2	1	29	1
9	10567	1.3	1	4	2	2	1	23	1
10	12093	9	7.17	1	2	2	1		1
11	3192	1.8	5.1	2	2	2	1	39	1
12	6606	5.7	0.9	2	2	2	1	27	1
13	4526	2.4	0.5	.	2	2	1	61	1
14	4901	5	0.5	4	2	2	1	19	1
15	7604	6.3	6.7	2	1	2	1	22	1
16	3346	4.2	1.9	3	1	2	1	23	1
17	5637	7.4	0.9	2	2	2	1	32	1
18	6894	8.1	2.8	1	2	2	1	29	1
19	6227	5.7	9.6	4	2	2	1		1
20	8811	4.7	0.8	1	1	2	1		1
21	5183	6.3	2.5	4	1	2	1	35	1
22	4510	5.3	7.6	.	1	2	1	23	2
23	4176	10.2	4.5	.	2	2	1	40	2
24	16210	13.1	2.9	4	2	2	1	20	2
25	3272	6.9	1.6	4	2	2	1	43	2
26	17760	4.4	6.4	3	2	2	1	45	2
27	6090	7	5	4	2	2	2	35	2
28	4048	28.8	4.05	4	2	2	1	59	2
29	3848	1.1	0.5	.	2	2	1		2
30	11600	6.16	1.5	4	2	2	1	32	2

EXCLI Journal 2026;25:330-338 – ISSN 1611-2156
Received: December 03, 2025, accepted: January 11, 2026, published: February 04, 2026

Code	ALT (U/L)	Bilirubin (mg/dL)	Creatinine (mg/dL)	INR	Sex (1= male, 2 = female)	Ethnicity (2 = Non-Hispanic)	Race (1 = White, 2 = Black, 4 = Asian)	Age	Died? (1 = No, 2 = Yes)
31	9151	7.9	3.21	4	2	2	1	44	2
32	3599	28.8	8.19	4	1	2	1	57	2
33	6796	6.8	2.43	4	2	2	1	27	2
34	4576	9.7	0.94	4	2	2	1	31	2
35	4613	21.7	6.06	4	1	2	1	27	2
36	4789	7.42	2.7	4	1	2	4	28	2
37	5510	11.1	3.14	4	2	2	1	29	2
38	8496	2.9	2.05	4	2	2	1	67	2
39	6528	5.7	4.54	4	2	2	1	36	2
